# Effects of hydrological regime and land use on in-stream *Escherichia coli* concentration in the Mekong basin, Lao PDR

**DOI:** 10.1038/s41598-021-82891-0

**Published:** 2021-02-10

**Authors:** Paty Nakhle, Olivier Ribolzi, Laurie Boithias, Sayaphet Rattanavong, Yves Auda, Saysongkham Sayavong, Rosalie Zimmermann, Bounsamay Soulileuth, Anne Pando, Chanthamousone Thammahacksa, Emma J. Rochelle-Newall, William Santini, Jean-Michel Martinez, Nicolas Gratiot, Alain Pierret

**Affiliations:** 1 Géosciences Environnement Toulouse (GET), Université de Toulouse, CNRS, IRD, UPS, Toulouse, France; 2 Lao-Oxford-Mahosot Hospital-Wellcome Trust Research Unit (LOMWRU), Microbiology Laboratory, Mahosot Hospital, Vientiane, Lao PDR; 3 Lao Department of Agriculture Land Management (DALaM), Ministry of Agriculture and Forestry, Vientiane, Lao PDR; 4 Department of Environmental Sciences, University of Basel, Basel, Switzerland; 5 Department of Medical Microbiology, Amsterdam University Medical Centers (UMC), Amsterdam, The Netherlands; 6 Institut de Recherche Pour le Développement (IRD), iEES-Paris, UMR 242 (IRD, SU-UPMC, CNRS, INRA, Univ. de Paris, UPEC), PO Box 5992, Vientiane, Lao PDR; 7 Institute of Ecology and Environmental Sciences of Paris (iEES-Paris), Sorbonne Université, Univ Paris Est Creteil, IRD, CNRS, INRA, Paris, France; 8 Univ. Grenoble Alpes, CNRS, IRD, Grenoble INP, IGE, 38000 Grenoble, France; 9 Asian Research Center on Water (CARE-Rescif), Ho Chi Minh City University of Technology, Block B7, 268 Ly Thuong Kiet Street, District 10, Ho Chi Minh City, Viet Nam

**Keywords:** Bacteria, Biogeochemistry, Environmental microbiology, Pathogens, Biogeochemistry, Environmental sciences, Hydrology, Gastrointestinal diseases

## Abstract

In the basin of Mekong, over 70 million people rely on unimproved surface water for their domestic requirements. Surface water is often contaminated with fecal matter and yet little information exists on the underlying mechanisms of fecal contamination in tropical conditions at large watershed scales. Our objectives were to (1) investigate the seasonality of fecal contamination using *Escherichia coli* as fecal indicator bacteria (FIB), and (2) establish links between the fecal contamination in stream water and its controlling factors (hydrology and land use). We present the results of (1) a sampling campaign at the outlet of 19 catchments across Lao PDR, in both the dry and the rainy seasons of 2016, and (2) a 10-day interval monitoring conducted in 2017 and 2018 at three point locations of three rivers (Nam Ou, Nam Suang, and Mekong) in northern Lao PDR. Our results show the presence of fecal contamination at most of the sampled sites, with a seasonality characterized by higher and extreme *E. coli* concentrations occurring during the rainy season. The highest *E. coli* concentrations, strongly correlated with total suspended sediment concentrations, were measured in catchments dominated by unstocked forest areas, especially in mountainous northern Lao PDR and in Vientiane province.

## Introduction

Emerging water quality issues resulting from various anthropogenic pressures and rapid global change are a topic of worldwide concern^1,2^. While the goal of improving surface water quality is being achieved in developed countries, an opposite trend towards increased organic and biological water pollution is observed in developing countries^3^. Fecal pollution of water resources is one of the major health issues in low-income tropical countries in Africa and Asia^3^, due to inadequate sanitation facilities, low access to safe water resources, and poor medical care. In 2015, diarrheal diseases caused more than 1.3 million deaths globally^4^. The World Health Organization recommends measuring fecal indicator bacteria (FIB), such as *Escherichia coli* (*E. coli*)*,* as a low-cost proxy for the presence of fecal pathogens in water bodies^5^. According to the 2016 UNEP report, about a third to one half of Asian rivers are estimated to be severely polluted, with monthly in-stream concentrations of fecal coliform bacteria, like *E. coli*, higher than 1000 cfu 100 mL^−1^. These large ranges of estimations reflect the uncertainties^3^ and stress the need to better assess at various space and time scales, the actual risks for public health in tropical areas associated with such pollutants.

During the last three decades, South-East Asia has experienced rapid economic development and population growth, strongly impacting water quality in many parts of the Mekong basin^6^. Commonly identified water quality issues in Mekong basin include microbiological pollution from human and animal waste, as well as chemical pollution from intensive agricultural activities leading to salinity and eutrophication increase^6^. In addition, the rapid hydropower development impacts the hydrological regime, sediment fluxes, and ecosystem services^7,8^. The Mekong is the third largest river in terms of water discharge in Southeast Asia^9^ and one of the most diverse aquatic ecosystems, making it the source of one of the largest inland fishery and aquaculture production system in the world^10^. In the lower Mekong basin stretching from southern Chinese border to the delta in southern Vietnam, over 70 million people depend on valuable ecosystem services provided by the transboundary Mekong river and its tributaries^10^. The livelihood and food security of rural populations are, as a consequence, highly vulnerable to changes in water quality.

Watersheds in Lao PDR contribute to approximately 35% of the overall Mekong river flow^10^. Like many developing countries, the population in Lao PDR is mostly rural^11^, and relies directly on untreated water via natural canal systems and small tributaries for domestic water use^12^. Diarrheal diseases are a leading cause of death nationwide, especially among children under age five^4,13^. However, no comprehensive survey of the fecal contamination of the Mekong, including main stream as well as tributaries and spanning successive seasons, has yet been published. Similarly, the underlying mechanisms of water fecal contamination has not been yet documented, although their understanding is needed to achieve an improved integrated management of water resources and to reduce associated diseases.

FIB dynamics in tropical waterbodies are subject to various interactive factors controlling their fate in the environment^14,15^. When FIB are released from hosts into secondary habitats (aquatic system and sediment), their persistence is function of abiotic factors including temperature, solar radiation, pH, salinity, nutrients, and suspended particles, and biotic factors like the presence of other microorganisms, biofilms and predators^14,16,17^. These factors can be strong drivers of microbial activity and stability in tropical context, potentially favorable for slower bacterial decay rates^18^. It has been shown that FIB tend to be associated with particles in soils and water columns^19,20^, which can provide benefits to microorganisms like access to nutrients, as well as protection from various stressors such as predation and sunlight^21,22^. In tropical regions, FIB is likely to be found correlated to total suspended sediment concentrations in rivers with high particle loads due to erosive tropical rainfall^20^.

Furthermore, several studies have documented FIB transport in the environment and shed light on the complex linkages between hydrometeorology and fecal contamination dynamics, both in urban and rural areas. During the rainy season, strong and frequent tropical rainfall and overland flow can induce declining surface water quality^23^, increased water turbidity, total suspended sediment and contaminant concentrations^12,24–26^. Deterioration of surface water microbial quality was highlighted by a study carried out in three tropical rural catchments in Lao PDR, Thailand and Vietnam, where the highest concentrations of *E. coli* in streams were found to occur during the rainy season^15^. Responses to tropical erosive rainfall are influenced by watershed topography and soil hydrodynamic properties (e.g*.,* drainage area, slope gradient, and infiltration rate), which are in turn strongly modulated by land use^25,27,28^. The scientific community has shown a growing interest in evaluating the impacts of the rapid land use change, including dam construction, that are affecting many parts of Southeastern Asia, with particular focus on the hydrological and sedimentary responses, at plot and hillslope scales^27,29–31^. Land use is considered to be one of the key factors controlling soil erosion^27,32^ over gentle to moderate terrain slope, and microbial community export, including *E. coli*, from the soil surface into the hydrographic networks in tropical environments^24,28,33,34^.

Although the main factors controlling the dynamics of *E. coli* are well evaluated in temperate and subtropical aquatic ecosystems^35–38^, only few studies have investigated the fate and transport of *E. coli* in tropical ecosystems. Key processes as well as environmental and anthropogenic drivers of *E. coli* dynamics were identified in small watersheds in tropical montane environments of Lao PDR^12,15,23,25,39–41^, of Singapore^26^, as well as in larger catchments, in northern Lao PDR^23,26^ and in the Mekong delta in Vietnam^18,42^.

While these studies have significantly improved our understanding on the behavior of FIB in tropical ecosystems on relatively local scales, the relevance of these findings on a regional-scale remains uncertain. Moreover, due to limited data in tropical developing countries, little information exists on long-term seasonal variations of FIB in large-scale watersheds, and none exists on the spatial distribution of FIB at the country scale in Lao PDR.

Our study is based on two sets of physico-chemical, microbiological, and environmental data. The first dataset was collected in 2016 during both the rainy and the dry seasons, in the Mekong river and 19 tributaries, in order to capture the spatial heterogeneity in terms of *E. coli* concentrations in the hydrographic network across Lao PDR. This approach was completed by a second dataset, a close-up investigation at 10-day interval between July 2017 and December 2018, of water quality in three watersheds at three point locations of three rivers (Nam Ou, Nam Suang, and Mekong) in northern Lao PDR, to provide better understanding of the FIB seasonal variations. The main objectives of this work were to (1) examine the seasonality of FIB (*E. coli*) concentrations and its response to contrasted hydrological events and (2) identify the relative importance of several parameters such as total suspended sediment concentrations, land use, rainfall event characteristics, human and livestock population density, as controlling factors of *E. coli* in-stream concentration at large-catchment scale, during both the dry and the rainy seasons.

## Results

### Spatial surveys conducted during the 2016 dry and rainy seasons

#### Descriptive analysis of the datasets

**Figure 1:**
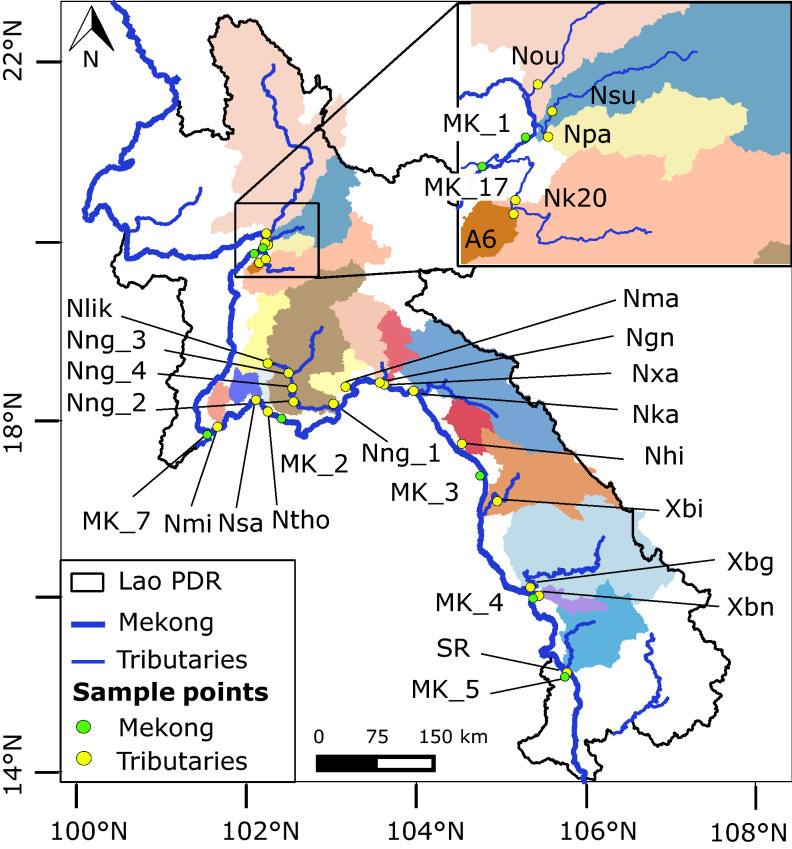
Mekong river and watersheds of Mekong tributaries (colored areas) sampled in March and July 2016 in Lao PDR. Geographic coordinate system: WGS 1984, latitude and longitude in degrees. Map generated with QGIS (version 2.6.1; https://www.qgis.org) and edited with Inkscape (version 0.92.4; https://inkscape.org).

During the March 2016 (dry season) and July 2016 (rainy season) sampling campaigns, we measured *E. coli* concentration ([*E. coli*]) and a set of physico-chemical parameters: turbidity; temperature (T); dissolved oxygen saturation (DO); pH; electrical conductivity (EC); total suspended sediment concentration ([TSS]); total nitrogen concentration ([TN]); and total particulate carbon concentration ([TPC]), in 19 Mekong tributaries (Fig. 1). The sampling sites were chosen to ensure a broad geographical coverage of Lao PDR, and to represent a large range of geographical, topographical, and land use features. The sampling sites were also chosen for being logistically accessible from the road, in order to cover the majority of Mekong tributaries in a relatively short time (Table 1, Table S1).

**Table 1:** Description of sampling sites in Lao PDR: names of river, geographical coordinates of sampling sites (i.e., latitude and longitude in degrees, WGS 1984)*,* sampling dates during field surveys in March and July 2016, and regular monitoring from July 2017 to December 2018, and catchment drainage area in km^2^.

Sampling sites	River	Geographical coordinates^a^		Sampling dates	Catchment area (km^2^)
		Latitude (°)	Longitude (°)		
Nou	Nam Ou	20.08642	102.26406	2016 2017–2018^b^	25,946
Nsu	Nam Suang	19.97931	102.24728	2016 2017–2018^b^	6577
Npa	Nam Pa	19.96049	102.28289	2016	758
MK_17	Mekong	19.89224	102.13416	2016 2017–2018^b^	27,215,525
Nk20	Nam Khan	19.78601	102.18335	2016	7236
A6	Houay Khan	19.76009	102.18112	2016	239
Nmi	Nam Mi	17.91917	101.68856	2016	1021
Nsa	Nam Sang	18.22284	102.14222	2016	1210
Ntho	Nam Thôn	18.09152	102.28159	2016	582
Nlik	Nam Lik	18.63280	102.28104	2016	3022
Nng_3	Nam Ngum	18.52502	102.52631	2016	8366
Nng_4	Nam Ngum	18.35581	102.57204	2016	14,318
Nng_2	Nam Ngum	18.20269	102.58588	2016	14,985
Nng_1	Nam Ngum	18.17879	103.05593	2016	16,841
Nma	Nam Mang	18.37017	103.19838	2016	1793
Ngn	Nam Gniep	18.41756	103.60217	2016	4564
Nxa	Nam Xan	18.39523	103.65408	2016	2223
Nka	Nam Kadin	18.32517	103.99924	2016	14,820
Nhi	Nam Hin Boun	17.72699	104.56798	2016	2152
Xbi	Xe Bang Fai	17.07782	104.98496	2016	9433
Xbg	Xe Bang Hieng	16.09804	105.37625	2016	19,817
Xbn	Xe Bang Nouan	16.00290	105.47937	2016	1351
SR	Nam Sedon	15.12390	105.80748	2016	7225
MK_1	Mekong	19.95601	102.24113	2016	26,388,036
MK_7	Mekong	17.89870	101.62397	2016	29,524,569
MK_2	Mekong	17.97276	102.50410	2016	30,182,592
MK_3	Mekong	17.39714	104.79999	2016	37,336,797
MK_4	Mekong	16.00503	105.42449	2016	41,709,405
MK_5	Mekong	15.10721	105.79878	2016	54,905,479

^a^Geographical coordinates in degrees (WGS 1984).

^b^Sampling frequency of 10-days interval.

**Figure 2:**
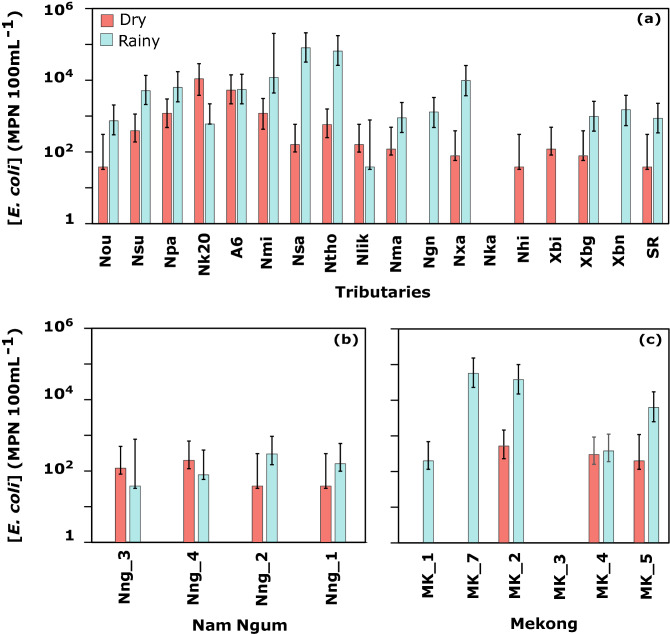
*E. coli* concentration with lower and upper 95% confidence limits ([*E. coli*], MPN 100 mL^−1^) in sampled watersheds of (**a**) Mekong tributaries, (**b**) Nam Ngum river, and (**c**) Mekong river across Lao PDR. Bars filled with orange represent concentrations during March 2016 (dry season) and bars filled with blue represent concentrations during July 2016 (rainy season). Sampling stations are classified from northern to southern Lao PDR. Figures generated with RStudio (version 1.2.1335; http://www.r-project.org) and edited with Inkscape (version 0.92.4; https://inkscape.org).

[*E. coli*] were found to be highly spatially variable across Lao PDR (Fig. 2), and 71% of the sampling sites displayed [*E. coli*] equal or greater to the lower detection limit of 38 MPN 100 mL^−1^ during both seasons. [*E. coli*] were below the detection limit in central and southern catchments (Nka, Ngn, Xbn) during the dry season and in central catchments (Nka, Xbi, Nhi) during the rainy season.

In March 2016, [*E. coli*] varied between 38 and 11,000 MPN 100 mL^−1^ at the outlet of the sampled Mekong tributaries (Fig. 2a). The highest [*E. coli*] were measured in four northern catchments (Nk20, A6, Npa, Nsu) and Vientiane plain (Nmi, Ntho), while the lowest [*E. coli*] were found in the mountainous parts of Vientiane Province (Nng_1), in central Lao PDR (Ngn), and in southern Lao PDR (Nka, Nhi, SR).

In July 2016, [*E. coli*] varied between 38 and 80,000 MPN 100 mL^−1^ at the outlet of the sampled Mekong tributaries (Fig. 2a). The highest [*E. coli*] were measured in the three catchments of the Vientiane plain (Nsa, Ntho, Nmi), and in catchments of northern Lao PDR (Npa, Nsu, A6).

Multiple sampling along the Nam Ngum river (Nng_3, Nng_4, Nng_2, and Nng_1), as well as along the Mekong river (MK_1, MK_7, MK_2, MK_3, MK_4, and MK_5), pointed out the spatial variability of *E. coli* contamination along rivers (Fig. 2b,c).

In March 2016, [*E. coli*] ranged between 38 and 200 MPN 100 mL^−1^ along the Nam Ngum river, decreasing in the downstream direction (Nng_1). In July 2016, [*E. coli*] ranged between 38 and 300 MPN 100 mL^−1^ along the Nam Ngum river, increasing in the downstream direction (Fig. 2b).

Moreover, [*E. coli*] were highly variable along the Mekong river mainstream (Fig. 2c). In the dry season, [*E. coli*] in the Mekong river ranged between 0 and 520 MPN 100 mL^−1^, while it varied between 0 and 57,000 MPN 100 mL^−1^ in the rainy season. The highest [*E. coli*] were found at the sampling sites located near urbanized areas around Vientiane (MK_7 and MK_2), and to a lesser extent in the southern station near Pakse (MK_5). The lowest [*E. coli*] were found in the highlands of northern Lao PDR (MK_1) and in southern Lao PDR (MK_3, MK_4) (Fig. 2c).

In the following statistical analyses on Mekong tributaries, only the Nam Ngum outlet (Nng_1) among the Nam Ngum sampling sites, was taken into account, to avoid the overrepresentation of the Nam Ngum tributary among the data.

**Figure 3:**
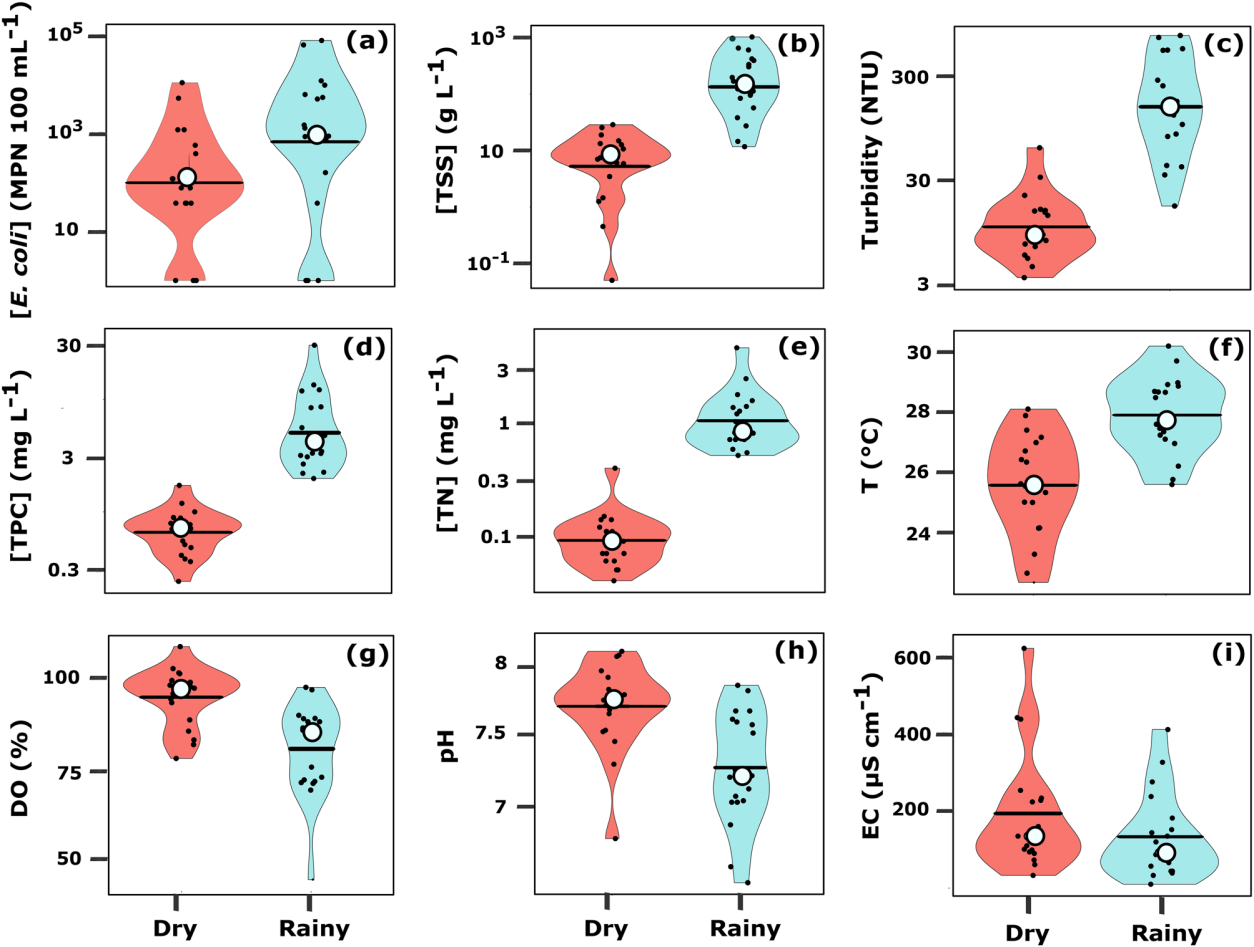
Violin plots of measured variables across sampling sites in Mekong tributaries of Lao PDR in March 2016 (dry season) and in July 2016 (rainy season): (**a**) *E. coli* concentrations ([*E. coli*], MPN 100 mL^−1^); (**b**) total suspended sediment concentrations ([TSS], g L^−1^); (**c**) turbidity (NTU); (**d**) total nitrogen concentration ([TN], mg L^−1^); (**e**) total particulate carbon concentration ([TPC], mg L^−1^); (**f**) temperature (T, °C); (**g**) dissolved oxygen saturation (DO, %); (**h**) pH; (**i**) electrical conductivity (EC, µS cm^−1^). Black circle represents the mean, the black line is the median, and black dots are the variable observations. Figures generated with RStudio (version 1.2.1335; http://www.r-project.org) and edited with Inkscape (version 0.92.4; https://inkscape.org).

Overall, seasonal variations of in-stream [*E. coli*] in Mekong tributaries were observed at the majority of the sampled sites. [*E. coli*] were different between the dry and the rainy seasons (p < 0.05), and followed a lognormal distribution during both seasons, yet more stretched towards upper extreme values during the rainy season (Fig. 3a).

Contrasted seasons also showed contrasted dynamics of physicochemical properties in the Mekong tributaries. [TSS], turbidity, [TPC], [TN], and T were significantly different between seasons (p < 0.05) (Fig. 3b–f). These variables followed the same trend as [*E. coli*], showing higher values during the rainy season compared to the dry season. DO, pH, and EC showed the opposite dynamics and were significantly higher during the dry season as compared to the rainy season (p < 0.05), with the exception of EC (Fig. 3g–i).

#### Partial Least Square (PLS) regression analysis

**Figure 4:**
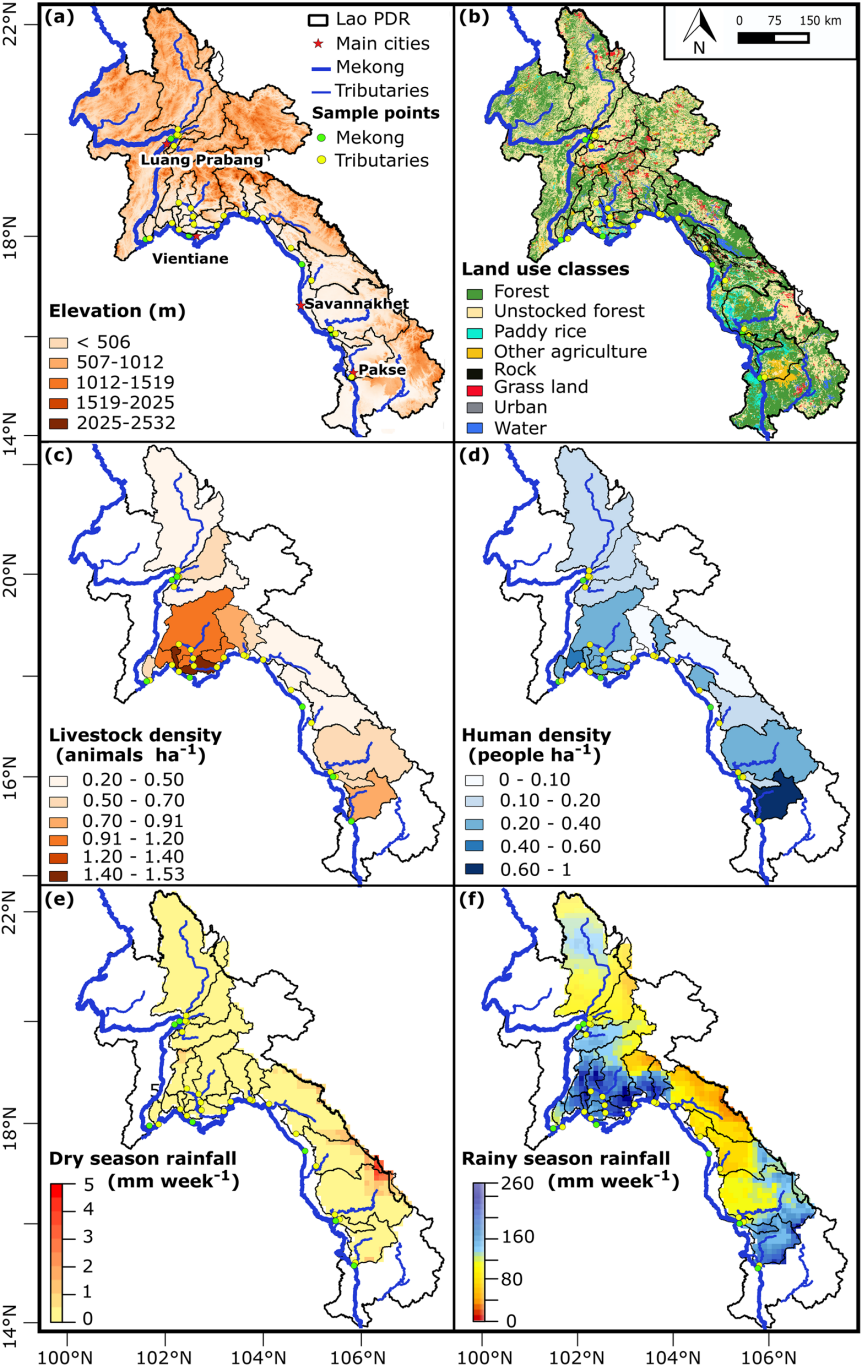
Geographical and meteorological characteristics of the sampled watersheds in Lao PDR: (**a**) geomorphological features (Digital Elevation Model); (**b**) land use classes; (**c**) livestock density; (**d**) human density; (**e**) total rainfall recorded one week pre-sampling in March 2016 (mm week^−1^); (**f**) total rainfall recorded one week pre-sampling in July 2016 (mm week^−1^). Geographic coordinated system: WGS 1984, latitude and longitude in degrees. Altitudes of highest and lowest points in meters above mean sea level, from SRTM 90 m. Local populations and livestock per district were taken from the Lao PDR Population and Housing Census 2015. Rainfall data were obtained from the Multi-Source Weighted-Ensemble Precipitation (MSWEP V2); spatially distributed rainfall data was averaged per sampled watershed area. The land use map included land cover classes, namely: rock, water, grassland, and forest, and land use classes, namely: unstocked forest, paddy rice, other agriculture, and urban areas, making up a total of eight classes. According to FAO 2010, forests refer to areas of more than 0.5 ha with a canopy cover of more than 10% and trees higher than 5 m. Unstocked forests are forests with crown density lower than 20% resulting from exploitation for logging or shifting cultivation. Unfertile or degraded areas covered by grass are attributed to grassland category. Other agriculture refers to agricultural lands used for non-crop purposes like livestock grazing. Water class includes rivers and water reservoirs exceeding 10 m of width and 0.5 ha of surface area. Urban areas include permanent settlements like villages, towns, and roads having a width of more than 5 m. Maps generated with QGIS (version 2.6.1; https://www.qgis.org) and edited with Inkscape (version 0.92.4; https://inkscape.org).

We conducted two separate PLS analyses aiming to identify the relative importance of the main controlling factors of [*E. coli*] at large-catchment scale, during each of the dry and the rainy seasons, in 19 Mekong tributaries. We considered the samples taken during the dry and the rainy seasons separately in order to discriminate the seasonal effect from the other factors (geomorphology, land use, population densities). We used constant parameters during 2016 such as altitude, slope, and catchment area, to describe the geomorphological features of these catchments (Fig. 4a). To describe the land use within the watershed, we used dams’ reservoir areas (Table S1), and areal percentages of unstocked forest, forest, paddy rice, grassland, urban, water and other agriculture areas in each catchment (Fig. 4b, Table S1), as well as human and livestock population densities per catchment (Fig. 4c,d, Table S1). Furthermore, we used seasonally variable parameters measured during both seasons in 2016, including [TSS], turbidity, [TN], [TPC], T, DO, pH, and EC; as well as the rainfall accumulated over one week before sampling (Fig. 4e,f).

**Figure 5:**
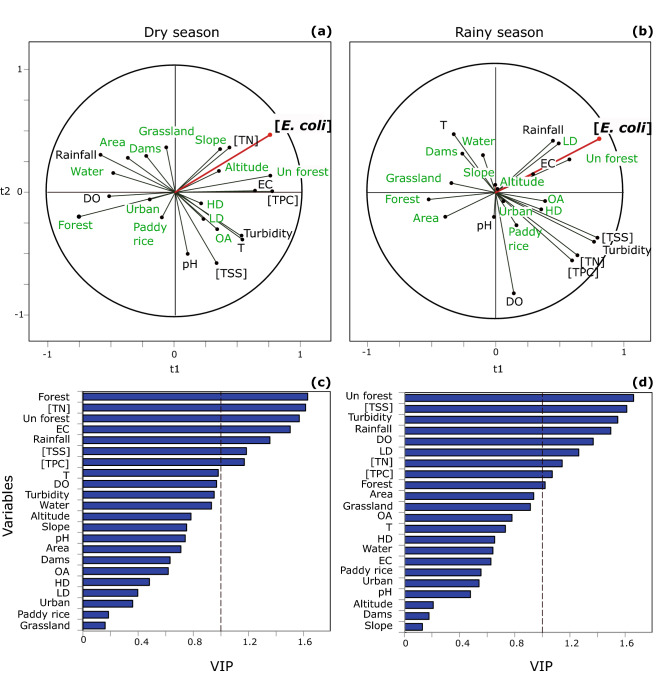
Correlation circles and variable importance in the projection (VIP) scores of the PLS regression analysis of variables measured in Mekong tributaries during the 2016 campaign: (**a**) correlation circle from PLS of dry season; (**b**) correlation circle from PLS of rainy season; (**c**) VIP plot from PLS of dry season; (**d**) VIP plot from PLS of rainy season. Variables in green are those that remain constant during 2016: catchment median altitude (Altitude, m), catchment median slope (Slope, %), catchment area (Area, ha), dams reservoir area (Dams, ha); human density (HD, people ha^−1^), livestock density (LD, animal ha^−1^), and percentage areas of unstocked forest (Un forest, %), forest (Forest, %), paddy rice (Paddy rice, %), grassland (Grassland, %), water (Water, %) and other agriculture (OA, %).Variables in black are those that were measured during both the dry and the rainy seasons: *E. coli* concentrations ([*E. coli*], MPN 100 mL^−1^); total suspended sediment concentration ([TSS], g L^−1^); turbidity (NTU); total nitrogen concentration ([TN], mg L^−1^); total particulate carbon concentration ([TPC], mg L^−1^); temperature (T, °C); dissolved oxygen saturation (DO, %); pH; electrical conductivity (EC, µS cm^−1^) and total rainfall recorded one week pre-sampling (Rainfall, mm week^−1^). Figures generated with XLSTAT (version 2020.5.1; https://www.xlstat.com) and edited with Inkscape (version 0.92.4; https://inkscape.org).

During the dry season, [*E. coli*] was mainly explained in the PLS model by the first component (57%) and to a lesser extent by the second component (21.5%) (Fig. 5a, Table S2). The VIPs (Variable Importance in the Projection) for each explanatory variable of both components showed that areal percentages of forest and unstocked forest, [TN], and EC, contributed the most to the model (VIP > 1.5) (Fig. 5c). During the rainy season, [*E. coli*] was mainly explained by the first component (66%) and to a lesser extent by the second component (19%) (Fig. 5c, Table S2). Areal percentages of unstocked forest, turbidity, and [TSS], were highly influential on the model (VIP > 1.5) (Fig. 5d).

During both seasons, the first component associated [*E. coli*] with common factors like watershed characteristics including areal percentages of land use classes, population density, catchment area, and physico-chemical parameters ([TSS], [TN], [TPC], and turbidity) (Fig. 5a,b). However, their relative importance changed with the season (Fig. 5c, d).

During both seasons, [*E. coli*] was positively correlated to unstocked forest percentage area (r = 0.65 in dry season, r = 0.62 in rainy season, p < 0.05) and negatively correlated to catchment area (r = − 0.49 in dry season, r = − 0.70 in rainy season, p < 0.05).

During the dry season, [*E. coli*] was positively correlated with EC (r = 0.59, p < 0.05) and T (r = 0.47, p < 0.05), and negatively correlated with forest percentage area (r = -0.55, p < 0.05) and DO (r = − 0.45, p = 0.054). During the rainy season, [*E. coli*] was positively correlated to [TN] (r = 0.48, p < 0.05), [TSS] (r = 0.44; p = 0.055), turbidity (r = 0.44, p = 0.060), [TPC] (r = 0.44; p = 0.061), and livestock density (r = 0.48, p = 0.054). Negative correlations were noted between [*E. coli*] and grassland percentage area (r = − 0.53, p < 0.05), and between [*E.* coli] and water percentage area (r = − 0.48, p < 0.05).

### Water quality monitoring of three northern watersheds during 2017 and 2018

**Figure 6:**
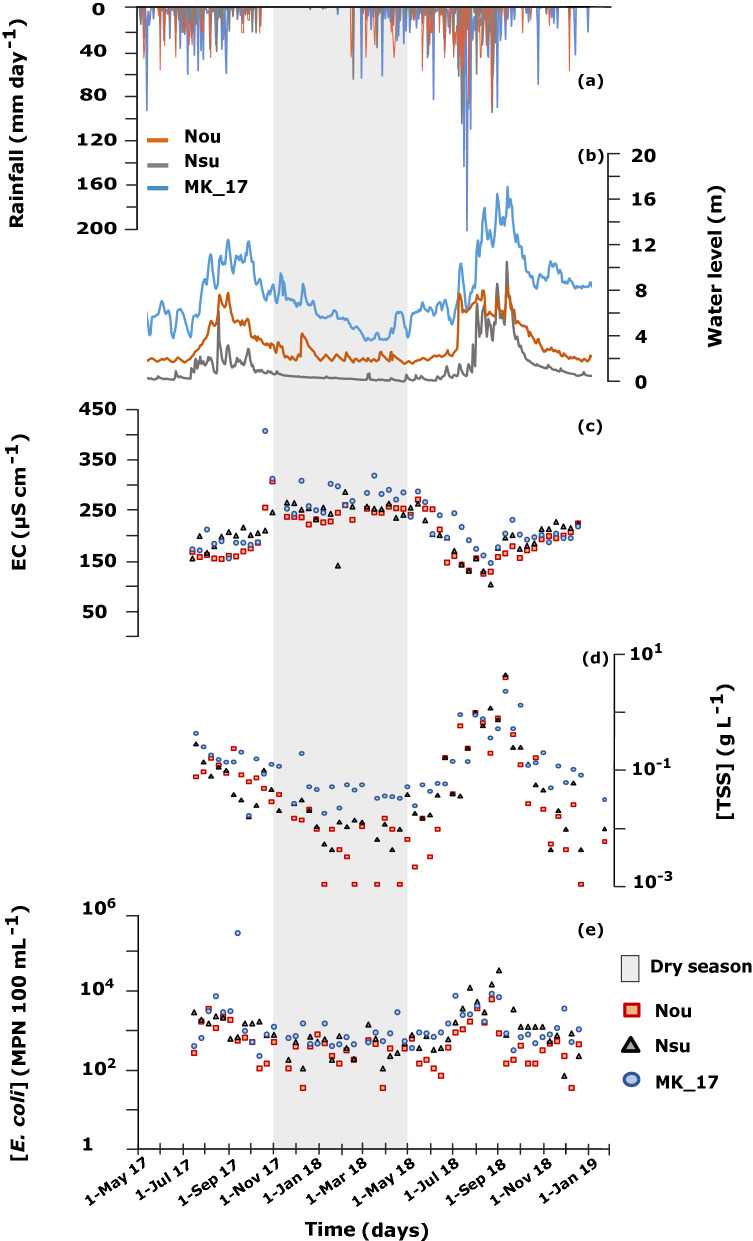
2017–2018 time series of variables measured at the outlet of three watersheds in northern Lao PDR, Nam Ou (Nou), Nam Suang (Nsu), and Mekong (MK_17): (**a**) daily rainfall (mm day^−1^) taken from meteorological stations of the Department of Natural Resources and Environment of Luang Prabang; (**b**) daily water level (m); (**c**) electrical conductivity (EC, µS cm^−1^); (**d**) total suspended sediment concentration ([TSS], g L^−1^); (**e**) *E. coli* concentration ([*E. coli*], MPN 100 mL^−1^). The highlighted area in grey represents the dry season (November–May). Figures generated with RStudio (version 1.2.1335; http://www.r-project.org) and edited with Inkscape (version 0.92.4; https://inkscape.org).

We conducted a closer investigation on *E. coli* dynamics through a 2-year water quality monitoring at 10-day intervals, at three point locations of three rivers in the vicinity of the city of Luang Prabang, northern Lao PDR: Nam Ou (Nou; 25,946 km^2^), Nam Suang (Nsu; 6577 km^2^), and Mekong (MK_17; 273,732 km^2^). The study period extended from July 2017 to December 2018, and spanned two rainy seasons, from May to October in 2017 and 2018 (Fig. 6). The periodic water sampling showed a continuous in-stream presence of *E. coli* in all of the three catchments over the 2017–2018 period. The highest range of [*E. coli*] was measured at the MK_17 sampling site on the Mekong river (250–350,000 MPN 100 mL^−1^), followed by the Nsu site at the outlet of Nam Suang (78–39,000 MPN 100 mL^−1^) and the Nou site at the outlet of Nam Ou (0–7100 MPN 100 mL^−1^). [*E. coli*] was marked by a seasonal variability, characterized by higher concentrations during the rainy season in all three watersheds (p < 0.05). [*E. coli*] was over two orders of magnitude and one order of magnitude higher during the rainy season compared to the dry season in both Nsu and Nou, and at MK_17, respectively.

Likewise, the seasonal variability of [TSS] in all sampled watersheds is marked by higher values during the rainy season (p < 0.05). [TSS] followed the same seasonal pattern as [*E. coli*], increasing over three orders of magnitude in Nsu, over two orders of magnitude in Nou, and over one order of magnitude at MK_17 during the rainy season. The opposite trend was noted for EC dynamics that showed higher values during dry season (p < 0.05). The lowest peaks of EC occurred during rainfall events and the highest values of EC were recorded during the dry season (Fig. 6). In Nou, Nsu, and MK_17, water level was positively correlated to [TSS] and [*E. coli*], and negatively correlated to EC (p < 0.05) (Table S3). Likewise, [*E. coli*] in all three watersheds was positively correlated to [TSS] and negatively correlated to EC (Table S3).

## Discussion

In this study, we investigated correlations between various environmental and water physico-chemical parameters and the occurrence of *E. coli*, as well as the seasonal variability of [*E. coli*] in the Mekong river and some of its major tributaries in Lao PDR.

Overall, our results show seasonal variabilities of [*E. coli*] and strong correlations between [*E. coli*] and [TSS] in the Mekong river and its tributaries. A seasonal comparison over two sampling years (2017–2018) have revealed contrasting hydrological, sedimental and microbiological responses that were driven by several factors. Higher water level, [TSS], and [*E. coli*], occurred in response to heavy rainfall. Similar distinctive seasonal fluctuations have been also observed in other tropical regions^18,42^. During the rainy season, surface runoff is a major factor controlling solid particles and *E. coli* transfer from top soil to the hydrographic network^24, 43–45^. This is consistent with our time series observations showing strong positive correlations between [*E. coli*] and [TSS] during rainy seasons, and negative correlations with EC values*.* The dynamics of EC can be used as an indicator of the relative contribution of overland flow to the streamflow^46,47^. A decreasing EC is characteristic of an increasing overland flow contribution, whereas an increasing EC indicates a higher contribution of groundwater^39,45^. During the 2017 and 2018 rainy seasons, EC decreased, suggesting an increase in overland flow contribution to streamflow, further contributing to the dispersion of suspended sediment and of microbial contaminants such as *E. coli* along hillslopes and downstream. Suspended sediment and washed-off free-living and particle-attached *E. coli* can deposit in streambeds of rivers before being re-suspended during high discharge events^39,48–50^. During the dry season, [*E. coli*] can be associated to other in-stream processes such as hyporheic exchange. In fact, during the dry season at baseflow, the stream-groundwater interactions through lateral flow and advective groundwater movement in the hyporheic zone may be responsible for remobilizing bacteria trapped in the porous space of streambed sediments^15,40^. Thus, the seasonal difference in terms of [TSS] and [*E. coli*] may be partly explained by seasonal streamflow regimes in response to meteorological patterns.

We noted similar trends in terms of positive correlations between [*E. coli*] and [TSS] during the rainy season of 2016 as compared to the rainy seasons of 2017 and 2018. Sources and dynamics of suspended particles were found to vary highly within a catchment (e.g., eroded sediments, re-suspended streambed sediments^18,39^). Suspended particles are known to be carriers of adsorbed pollutants, nutrients, and microorganisms like *E. coli*, partly controlling their transport and fate^18,19,51^. Furthermore, suspended particles are not only vectors for bacterial transport, they could also provide optimal conditions for the survival of adsorbed coliform bacteria by protecting them from ultraviolet radiation and predators^42–44^. Bacterial decay rates can be influenced by the physico-chemistry of the stream water (e.g., pH, dissolved oxygen saturation, turbidity, EC and salinity^18,52–54^). Water physico-chemical properties could be key drivers explaining the higher *E. coli* concentrations in the majority of the sampled tributaries during the rainy season.

In the present work, PLS analysis also helped to identify various correlations between land use classes and [*E. coli*] in stream water. During both dry and rainy seasons, [*E. coli*] was positively associated with the percentage of unstocked forest area and negatively associated with the percentage of forest and grassland areas, especially during the rainy seasons. Such correlations are consistent with reports from previous studies in similar tropical watersheds^14,26,33,55^. Higher FIB concentrations were measured in watersheds located in steep mountainous areas of northern Lao PDR as well as in the Vientiane plain, where the dominant land use is unstocked forest, and to a lesser extent, paddy rice and other agricultural land. The unstocked forest percentage areas are largely present in Southeast Asian upland catchments, due to the general pressure towards clearing forests for intensified annual crop production with reduced fallow period and suppressed understory cover^27,34,49,56^. Forest areas in Lao PDR have shrunk from about 73% in the 1960s to 40% in the 1990s^33,57,58^. These land use changes can affect soil erosion, water infiltration and overland flow^33,59–61^, due to reduced topsoil cover and reduced soil binding by roots^33,62^. In steep regions, deforestation may also lead to landslides due to the absence of stabilizing roots network and loss of soil stability^58,60^. Unstocked forest areas are thereby more vulnerable to soil erosion and landslides processes^29,63^, especially during the rainy season, increasing overland flow loaded with soil particles and attached FIB that end up in the downstream river.

In contrast, southern Lao PDR watersheds (Nka, Nhi, Xbi), characterized by high percentages of forest areas, had the lowest contamination during both seasons. Areas dominated by forested watersheds with understory cover have been shown to have high infiltration rates, low soil erosion, and high contaminant trapping efficiencies^64,65^. Land cover and management practices can be key factors controlling runoff production and surface soil erosion, and thus FIB contamination levels of rivers^52,66^.

In our PLS analyses, negative associations between [*E. coli*] and dams’ reservoir area were noted during both seasons, yet they were not significant at the 0.05 level in our study case. The impact of dams on FIB could be different in each catchment, depending on dams’ reservoir area, as well as on the distance between dams and sampling sites and on potential FIB sources in-between. Many studies focused on the potential hydrological effects of dams in the Mekong basin^67^. An important decrease in suspended sediment loads was noted in many tributaries following the construction of dams, for instance a 50% reduction at Pakse where the average suspended loads decreased from 120 to 60 Mt yr^−1^^68^. Along with flow alteration and sediment trapping, dams could also impact FIB fate and transport.

*Escherichia coli* sources vary greatly across Lao PDR, depending on human and animal density, as well as on the presence or absence of an operational wastewater collection system. It has been shown that mammalian presence closely influences the river’s microbiological quality, particularly in rural areas of developing countries lacking sanitary infrastructure^15,24^. However, our study did not show any significant correlation between human densities and measured downstream [*E. coli*] in either the dry or the rainy seasons. Livestock densities were weakly correlated with [*E. coli*] during the rainy season (r = 0.45, p = 0.054). This can tentatively be ascribed to several reasons.

On the one hand, the population of Lao PDR is unevenly distributed across the country. About 70% of the population lives in rural areas. Additionally, there are significant urban–rural disparities in terms of access to improved sanitation facilities. Thus, some rural watersheds, although less densely populated, are more exposed to fecal contamination through point sources (direct release of untreated wastewater into river stream), as compared to other populated urban watersheds equipped with wastewater treatment systems. However, despite incremental improvements of the sanitation system in Lao PDR, open defecation remains a major issue, estimated to concern 32% and 45% of the overall and rural populations, respectively^69^. Along with the presence of wild animals or livestock, open defecation is a diffuse source of microbial pathogens, transferred to the stream with surface runoff, and contributing to the fecal pollution of rivers. Moreover, unstocked exploited forests in watersheds with low mammalian presence can be highly frequented by workers during specific periods and by villagers for their domestic needs, adding to the complexity of determining diffuse source inputs. On the other hand, the absence of strong correlations between human and livestock densities and FIB concentrations may also be due to the distance between primary sources (human and livestock) and water streams. This distance, in turn, affects survival rates of transferred FIB, which get increasingly exposed to environmental conditions as primary sources and streams are more distant from one another. During hot and dry periods, less favorable conditions for microbial development (e.g. more sunlight, less nutrients) might increase opportunities for their die-off^14,18,70^. In addition, longer transfer times from hillslopes to rivers due to disconnected flow paths might result in lower [*E. coli*] in downstream rivers due to the sedimentation of FIB-bound particles and FIB decay^55,71^. However, *E. coli* might be able to survive in streambed sediment reservoirs, explaining a continuous presence of *E. coli* in streams, even during the dry season. Several laboratory experiments and field studies have shown that streambed sediments, under favorable conditions, are important bacterial reservoirs, both in temperate regions^72–74^ and tropical conditions^39,75^.

PLS analyses helped identifying the relative importance of variables explaining [*E. coli*] in 19 tributaries across Lao PDR in dry and rainy season. While this study provides useful insights on relationships between various factors and FIB, we were limited by the available data, especially the land use data. In future studies, when updated land use data will be available, it will be necessary to differentiate between planted and natural forests within the forest category at watersheds scale. The impacts of commercial tree plantations (e.g., teak trees, rubber trees) on hydrological response and associated increase in soil erosion, were pointed out by a few studies^28,33,76^. More accurate, complete, and higher land use data resolution would allow a closer understanding of the factors controlling bacterial contaminations and should be taken into account when addressing land management issues.

## Conclusion

This study is the first to assess seasonal dynamics of fecal contamination in Lao PDR, based on a large physico-chemical, microbiological, and geomorphological dataset, with the aim of identifying the relative importance of different controlling factors of in-stream *E. coli* concentrations during both the dry and the rainy seasons. Our study consisting of (1) a spatial survey in 2016 during both the dry and the rainy seasons, and (2) a 10-day sampling monitoring from July 2017 to December 2018 at 3 stations, pointed out the following main findings.

- The seasonal variability of *E. coli* concentrations marked by higher and extreme values occurring during the rainy season, is noted in the majority of sampled Mekong tributaries. This is consistent with the increase in surface water turbidity during rainy season.
- *E. coli* concentrations are positively correlated to total suspended sediment concentrations in both of the datasets, highlighting the potential role of suspended sediment dynamics in FIB transport, more particularly in catchments prone to soil erosion in a tropical setting.
- *E. coli* concentrations are positively correlated with unstocked forest percentage areas, and negatively correlated to forest percentage areas, which points out the importance of land use/land cover as one of key factors impacting FIB dynamics at catchment-scale.

Our data provide new evidence that populations relying on untreated surface water resources of three northern watersheds in Lao PDR (Nam Ou, Nam Suang, and Mekong) are exposed to continuous fecal contamination all year round. Despite the World Health Organization (WHO) guidelines of 0 MPN 100 mL^−1^ of *E. coli* in drinking water, this remains an urgent public health issue in Lao PDR, putting lives at risk. The majority of sampled tributaries across Lao PDR in 2016 presented very high *E. coli* concentrations during the rainy season, exceeding 500 colonies per 100 mL, the threshold above which the WHO considers a 10% risk of gastrointestinal illness after one single exposure. This stresses the need for a better water quality assessment of the Mekong river and its tributaries, as well as a detailed evaluation of the risks posed by fecal waterborne diseases to rural populations directly depending on untreated water resources. In addition, given the rapid growth of hydropower plants in the Mekong basin^7, 8^, it will be necessary to investigate its impact on hydrology, sediment fluxes, and associated health issues like fecal contamination at watershed-scale in Lao PDR. It is also necessary to further investigate and quantify the factors controlling FIB survival and mortality in water and sediments under tropical conditions to understand the dynamics of these bacteria in this system.

## Material and methods

### Study site characteristics

Sampling sites were located in Lao PDR, a landlocked country in Southeast Asia, sharing borders with Myanmar, Cambodia, China, Thailand, and Vietnam. Lao PDR is mainly covered with mountains and forested hills, plateaus and plains along the Mekong river, where approximately 6.5 million people live on a 236,800 km^2^ land^11^. About 70% of its population lives in rural areas and have a resource-based economy, relying mainly on agriculture and forestry for their livelihood. The tropical wet and dry climate (Aw climate) is under the influence of monsoon regime, dividing the year into two seasons: a dry season from October to April, and a rainy season from May to October. The average annual rainfall in Lao PDR varies from 1300 to 2500 mm and exceeds 3500 mm in central and Southwestern Lao PDR (Fig. S1). The air temperature ranges from a minimum 15 °C in December-January to a maximum temperatures of 25 ± 30 °C from May to September^23^. The Mekong river runs about 4350 km through China, Lao PDR, Myanmar, Thailand, Cambodia and Vietnam, draining a 795,000 km^2^ surface area^77^. The river flows from North to South of Lao PDR, forming a natural border with Thailand over 800 km.

### Sampling design and watersheds characteristics

Our study investigates seasonal dynamics of fecal contamination, based on field monitoring at multiple space and time scales. We used two different datasets (Fig. 1; Table 1).

1. A field campaign conducted in 2016 that sampled the Mekong river at six sampling sites, and 19 Mekong tributaries at 22 sampling sites including four sites along the Nam Ngum river (Fig. 1) located on mountains, hills and plains (Fig. 4a). The sampling was conducted once in March 2016 (dry season) and once in July 2016 (rainy season). The choice of sampling sites was based on a broad geographical coverage of Lao PDR between 15 and 20°N, and to encompass a broad range of catchment sizes (239–25,946 km^2^), and a large range of geographical, topographical, and land use features. The sampling sites were also chosen to allow a large spatial sampling, covering the majority of the Mekong tributaries, in a relatively short time and logistically accessible from the road (Table 1, Table S1).
2. Regular water quality monitoring of three rivers in northern Lao PDR, conducted at 10-day intervals from July 2017 to December 2018, including two rainy seasons from April to October 2017 and 2018: two sampling points on the Nam Ou (Nou) and Nam Suang (Nsu), both located on the left bank of the Mekong river, and one sampling point in the Mekong river in Luang Prabang (MK_17).

The surface area of the catchments of the Mekong tributaries ranges from 239 km^2^ (A6) to 25,946 km^2^ (Nou) (Table 1). A range of eight different land use classes, grouped in eight main categories, is found across Lao PDR (Fig. 4b; Table S1). The highest percentage of unstocked forest areas and the lowest percentage of forest areas were found in catchments of northern Lao PDR (Nou, Nsu, Npa, Nk20, and A6), and Vientiane Province (Nmi, Nsa, and Ntho), whereas forest areas dominated the catchments in southern Lao PDR (Nka, Nhi, Xbi, Xbg, Xbn, and SR). The highest percentages of paddy rice and other agriculture areas were found in the southern catchment near Pakse (SR) followed by catchments in Vientiane Province (Nmi, Nsa, and Ntho). The highest percentages of grassland and water areas were found in Vientiane Province (Nlik, Nng_3, Nng_4, Nng_2, and Nng_1). The livestock population is mostly present in catchments near Vientiane Capital followed by southern provinces of Savannakhet and Pakse (Fig. 4c; Table S1). Human population density is variable across Lao PDR. The densest catchments are mostly found in the southern province of Pakse, followed by Savannakhet and Vientiane Capital (Fig. 4d, Table S1). Catchments are also highly variable in terms of rainfall (Table S1). During March 2016 (dry season), the rainfall was low (Fig. 4e). During July 2016 (rainy season), the highest rainfall was recorded in catchments located on steep terrain (Nlik, Nng_3, Nng_4, Nng_2, and Nng_1) and plains (Nmi, Nsa, and Ntho) of Vientiane Province (Fig. 4f; Table S1).

### Geographical analysis

The SRTM 90-m resolution digital elevation model (DEM) was used to draw the elevation map in the QGIS 2.6.1 software (https://www.qgis.org/en/site/forusers/). Based on this DEM and the geographical location of the sampling points, the catchment areas were then determined using the QGIS 2.6.1 software. We also computed surface area, perimeter, median slope, and median elevation of each of the catchment areas upstream of the sampling points from the DEM (Fig. 4a).

### Data on land use and dams

We used information from the land use map provided by the Department of Agriculture Land Management (DALaM) of Lao PDR in 2013 (Fig. 4b). Technically, this land use map included land cover classes, namely: rock, water, grassland, and forest, as well as land use classes, namely: unstocked forest, paddy rice, other agriculture, and urban areas, making up a total of eight classes expressed in percentage of surface area. According to Lao Forestry Administration^78^ and the Food and Agriculture Organization of the United Nations (FAO)^57^, forests refer to areas of more than 0.5 ha with a canopy cover of more than 10% and trees higher than 5 m. Unstocked forests are forests with a crown density lower than 20% resulting from exploitation for logging or shifting cultivation cycles. Unfertile or degraded areas covered by grass are attributed to grassland category. Other agriculture refers to agricultural lands used for non-crop purposes like livestock grazing. Water class includes rivers and water reservoirs exceeding 10 m of width and 0.5 ha of surface area. Urban areas include permanent settlements like villages, towns, and roads having a width of more than 5 m. Data on regional dams in Lao PDR represent maximum reservoir area (ha) of dams located upstream of our sampling sites. These data were taken from the dataset on the dams of the greater Mekong by Mekong Region Futures Institute^79^.

### Data on livestock and local populations

We obtained data on local populations and livestock per district from the Lao PDR Population and Housing Census 2015 conducted by Lao PDR Statistics Bureau^11^. Based on the percentage of area occupied by each district present in each catchment, we calculated the density of the human population and of livestock in each basin's catchment area (Fig. 4c,d). The data on livestock densities (cattle, buffaloes, pigs, goats, ducks, local and commercial chickens) were obtained from the Lao agricultural census 2010/2011^80^.

### In situ measurements and laboratory analysis

We measured a set of physico-chemical parameters in situ, including stream water temperature (T), pH, electrical conductivity normalized to 25 °C (EC), dissolved oxygen saturation (DO), using a Multi Probe System with a data logger (YSI 556 MPS). Concentrations of DO were transformed to oxygen saturations (%), using the Hua formula^81^. We collected 500-mL water samples 10 cm beneath the water surface in sterile plastic containers, and conserved them at a low temperature in the dark until laboratory analysis. A subsample of the collected water was analyzed within 24 h, for *E. coli* counts, using the standardized microplate method (ISO 9308–3). The latter consists of a 48-h incubation at 44 °C of each sample, at four dilution rates (1:2, 1:20, 1:200 and 1:2000), in a culture medium specific for *E. coli* on a 96-well microplate (MUG/EC, Biokar Diagnostics). By counting the number of positive wells for each microplate, and applying the Poisson distribution, the index of viable bacteria called most probable number (MPN) can be determined. The concentration of *E. coli* ([*E. coli*]) is expressed in MPN 100 mL^−1^ and used as an indicator of the fecal contamination^82^. This method has already been used successfully in a tropical context^15,24^. Another subsample was analyzed for total suspended sediment concentration ([TSS]): [TSS] was determined for each sample after filtration on 0.2 μm porosity cellulose acetate filters (Sartorius) and evaporation in an oven at 105 °C for 48 h. The concentrations of total particulate carbon ([TPC]) and of total nitrogen ([TN]) in river water (mg L^−1^), were determined by isotope ratio mass spectrometry coupled with elemental analyzer (Integra 2 Stable Isotope Analyser, Sercon).

### Rainfall and water level

We used the Multi-Source Weighted-Ensemble Precipitation (MSWEP V2) 2016 rainfall data in order to have accurate, spatially distributed precipitation over the watersheds of interest. MSWEP is a global precipitation product merged from gauge, satellite, and reanalysis data^83^. It has a high temporal (3-hourly) and spatial (0.1°) resolution, which was evaluated and assessed on a global scale^84^ and in Eastern Asia^85,86^. In our study, we used MSWEP cumulative rainfall data over a 1-week period (corresponding to the sampling campaign duration) before the sampling during the 2016 campaign, averaged per sampled watershed area.

The daily rainfall measurements for 2017 and 2018 associated with the Mekong, Nam Ou, and Nam Suang sampling sites, were taken from three stations of the Lao PDR Government (Department of Natural Resources and Environment of Luang Prabang). The rainfall station in Nam Ou (20.081972, 102.264139) is located at the outlet of the catchment, at a 530 m distance from the stream sampling point, in Nam Suang (19.967111, 102.272444) at 3 km upstream to the sampling point, and in Mekong (19.898472, 102.16525) at 3 km distance from MK_17 sampling point. Water level data were measured at three gauging stations situated in Nam Ou (20.245556, 102.348806) at 21 km upstream the outlet, in Nam Suang (19.967111, 102.272444) at 3 km upstream the sampling point, and in Mekong (19.892361, 102.134167) near MK_17.

### Statistical analysis

In this study, we first focused on identifying links between [*E. coli*] and discriminating variables like hydro-meteorological factors, land use, and geomorphological characteristics of 19 Mekong tributaries in Lao PDR. Therefore, we used an exploratory data analysis, namely the Partial Least Square (PLS) regression, which is a multivariate approach^87^. It is the most adapted to our dataset, as it is able to handle (1) a large database with more variables than observations, (2) nonlinear relationships between response and independent variables, and (3) possibly collinear variables^88^. Before applying the PLS algorithm, we centered and normalized the data, since the dataset included variables with different scales and measurement units. Moreover, turbidity, [TSS], and [*E. coli*], were log-transformed because the measured data were positively skewed. The variable importance in the projection number (VIP) was computed to determine the importance of variables. VIP greater than 1 are considered to be important in the analysis. The PLS analyses were done using the Microsoft Excel for Windows 2016 add-ins with XLSTAT version 2020.5.1^89^.

To test the significant difference between measured variables in Mekong and Mekong tributaries during dry and rainy seasons, we divided the 2017–2018 monitoring dataset into two datasets: a dry season from November to May, and a rainy season from June to October. We used the Wilcoxon-test, to test the significant difference of measured variables in 19 tributaries between dry and rainy seasons of the 2016 campaign, and of the 2017–2018 monitoring datasets, with statistical significance set at p-value < 0.05.

We calculated Spearman correlation coefficients to investigate relationships between measured variables and [*E. coli*] measured in 19 tributaries during the 2016 campaign, as well as during the water quality monitoring 2017/2018 at the outlet of three watersheds in northern Lao PDR (Nam Ou, Nam Suang, Mekong). In the statistical analyses on Mekong tributaries (PLS analysis, Spearman correlation and Wilcoxon test), only the Nam Ngum outlet (Nng_1) among the Nam Ngum sampling sites, was taken into account, to avoid the overrepresentation of the Nam Ngum tributary among the data. The Wilcoxon-test and the Spearman correlations were conducted using RStudio version 1.2.1335^90^. The original maps were created using QGIS 2.6.1^91^.

## Supplementary Information

Supplementary Information.

## Data Availability

The datasets generated during and analysed during the current study are available from the corresponding author on reasonable request.
