# Supplementary material for: Effects of hydrological regime and land use on in-stream Escherichia coli concentration in the Mekong basin, Lao PDR
Source: Sci Rep. 2021 Feb 10;11:3460. doi: 10.1038/s41598-021-82891-0 (PMC7876097; doi:10.1038/s41598-021-82891-0)
Supplement: Supplementary file 1 — Supplementary Information. [file 41598_2021_82891_MOESM1_ESM.pdf]

Supplementary Information

Effects of hydrological regime and land use on in-stream *Escherichia coli* concentration in the Mekong basin, Lao PDR

Paty Nakhle[1], Olivier Ribolzi[1], Laurie Boithias[1,*], Sayaphet Rattanavong[2], Yves Auda[1], Saysongkham Sayavong[3], Rosalie Zimmermann[2,4,5], Bounsamay Soulileuth[6], Anne Pando[6], Chanthamousone Thammahacksa[6], Emma J. Rochelle-Newall[7], William Santini[1], Jean-Michel Martinez[1], Nicolas Gratiot[8,9] & Alain Pierret[6]

[1] Géosciences Environnement Toulouse (GET), Université de Toulouse, CNRS, IRD, UPS, Toulouse, France;
[2] Lao-Oxford-Mahosot Hospital-Wellcome Trust Research Unit (LOMWRU), Microbiology Laboratory, Mahosot Hospital, Vientiane, Lao PDR;
[3] Lao Department of Agriculture Land Management (DALaM), Ministry of Agriculture and Forestry, Vientiane, Lao PDR;
[4] Department of Environmental Sciences, University of Basel, Basel, Switzerland;
[5] Department of Medical Microbiology, Amsterdam University Medical Centers (UMC), Amsterdam, The Netherlands;
[6] Institut de Recherche pour le Développement (IRD), iEES-Paris, UMR 242 (IRD, SU-UPMC, CNRS, INRA, Univ. de Paris, UPEC), PO Box 5992, Vientiane, Lao PDR;
[7] Institute of Ecology and Environmental Sciences of Paris (iEES-Paris), Sorbonne Université, Univ Paris Est Creteil, IRD, CNRS, INRA, Paris, France;
[8] Univ. Grenoble Alpes, CNRS, IRD, Grenoble INP, IGE, F-38000, Grenoble, France;
[9] Asian Research Center on Water (CARE-Rescif), Ho Chi Minh City University of Technology, Block B7, 268 Ly Thuong Kiet Street, District 10, Ho Chi Minh City, Viet Nam.
* Corresponding author: laurie.boithias@get.omp.eu

Table S1: Summary of geographical and meteorological characteristics per group of sampled watersheds in Lao PDR. Altitude (m a.m.s.l.), slope (%), areal percentages of: forest (%), unstocked forest (%), paddy rice (%), other agriculture (OA, %), grassland (%), water (%), and urban (%), dams' reservoir area (Dams, ha), human density (HD, people ha^{-1}), livestock density (LD, animal ha^{-1}), rainfall during rainy season (mm $week^{-1}$) one week preceding the sampling in July 2016.

Sampling sites	Altitude	Slope	Forest	Unstocked forest	Paddy rice	OA	Grass-land	Water	Urban	Dams	HD	LD	Rainfall rainy season
	(m a.m.s.l.)	(%)	(%)	(%)	(%)	(%)	(%)	(%)	(%)	(ha)	(people ha^{-1})	(animal ha^{-1})	(mm $week^{-1}$)
**Nou**	887	30	30.7	56.6	0.1	2.9	2.7	1.4	0	1,570	0.2	0.4	108
**Nsu**	839	32.7	21.9	72.9	0	1.5	3.3	0.2	0	0	0.1	0.5	88
**Npa**	900	33.9	13.9	78.9	0.1	3.6	3.6	0	0	0	0.2	0.6	107
**Nk20**	950	31.6	31.8	56.3	0	3.3	8.4	0.1	0	3,056	0.2	0.5	124
**A6**	820	30.3	18.8	74	0.6	4.4	2.2	0	0	0	0.3	0.8	128
**Nmi**	345	13.9	31	47.1	4.8	16.6	0.3	0	0	0	0.2	0.6	128
**Nsa**	325	13.5	33.3	56.7	1.8	8	0	0.1	0	0	0.4	0.9	175
**Ntho**	235	5.62	25.6	60.1	9.5	4.7	0	0.2	0	0	0.3	1.5	206
**Nlik**	522	24.1	51.5	33.8	2.3	6.7	4.6	1.1	0	24,40	0.2	0.7	175
**Nng_1**	631	18.3	33	41.6	5.2	7.8	6.9	5.1	0.3	37,000	0.4	1.5	161
**Nma**	596	14.2	49.9	41.4	1.1	1.4	3.5	1.8	0.1	1,720	0.1	0.9	203
**Ngn**	990	25.4	31.8	51.5	0.6	4.1	7.6	4.4	0	0	0.1	0.7	117
**Nxa**	373	21.5	55.6	39	2.2	1.8	0.8	6.5	0	12,300	0.2	0.6	143
**Nka**	707	24.2	60.2	28	0.5	1	2.9	0.5	0	0	0.1	0.3	69
**Nhi**	310	16	31.1	27.6	5.3	1.1	2.7	1.7	0	0	0.2	0.4	83
**Xbi**	246	8.6	44	32.1	5.5	1.7	3.4	0.3	0	0	0.2	0.4	97
**Xbg**	237	4.7	44.9	36.8	9	1.9	2.3	1.2	0	0	0.3	0.5	126
**Xbn**	278	5.4	70.2	23.5	3	1.1	1.6	0.5	0	0	0.1	0.7	152
**SR**	244	3.4	44.9	17.2	16.9	19.5	0.9	0.3	0.2	55	0.7	0.9	172

Table S2: Model quality by number of components, the first component (Comp1) and the second component (Comp2) of the PLS analyses for dry and rainy seasons. The Q^2 cumulated (Q^2 cum) index measures the global contribution of the first two components to the predictive quality of the model. The R^2Y cumulated (R^2Y cum) index represents the sum of the coefficients of determination between the dependent variables and the two first components. The R^2X cumulated (R^2X cum) index is the sum of the coefficients of determination between the explanatory variables and the two first components.

	**Dry season**	
**Statistics**	**Comp1**	**Comp2**
**Q^2 cum**	0.290	-0.297
**R^2Y cum**	0.568	0.787
**R^2X cum**	0.208	0.291
	**Rainy season**	
**Statistics**	**Comp1**	**Comp2**
**Q^2 cum**	0.481	0.477
**R^2Y cum**	0.657	0.841
**R^2X cum**	0.178	0.292

Table S3: Spearman correlation coefficients between *E. coli* concentration ([*E. coli*], MPN 100 mL^{-1}), total suspended sediment concentration ([TSS], g L^{-1}), electrical conductivity (EC, µS cm^{-1}), water level (m), and rainfall (Rainfall, mm day^{-1}), measured from July 2017 to December 2018 at the outlet of three watersheds in northern Lao PDR: Nam Ou (Nou), Nam Suang (Nsu), and Mekong (MK_17). Values in bold letters indicate significant correlation ($p < 0.05$).

		**Nou**		
	[*E. coli*]	[TSS]	EC	Water level
[TSS]	**0.45**	-		
EC	**-0.55**	**-0.76**	-	
Water level	**0.52**	**0.86**	**-0.82**	-
Rainfall	0.24	0.25	-0.17	0.25
		**Nsu**		
	[*E. coli*]	[TSS]	EC	Water level
[TSS]	**0.66**	-		
EC	**-0.72**	**-0.68**	-	
Water level	**0.72**	**0.77**	**-0.85**	-
Rainfall	**0.62**	**0.51**	**-0.50**	**0.49**
		**MK_17**		
	[*E. coli*]	[TSS]	EC	Water level
[TSS]	**0.44**	-		
EC	**-0.30**	**-0.60**	-	
Water level	**0.48**	**0.71**	**-0.79**	-
Rainfall	**0.31**	**0.41**	**-0.33**	**0.36**

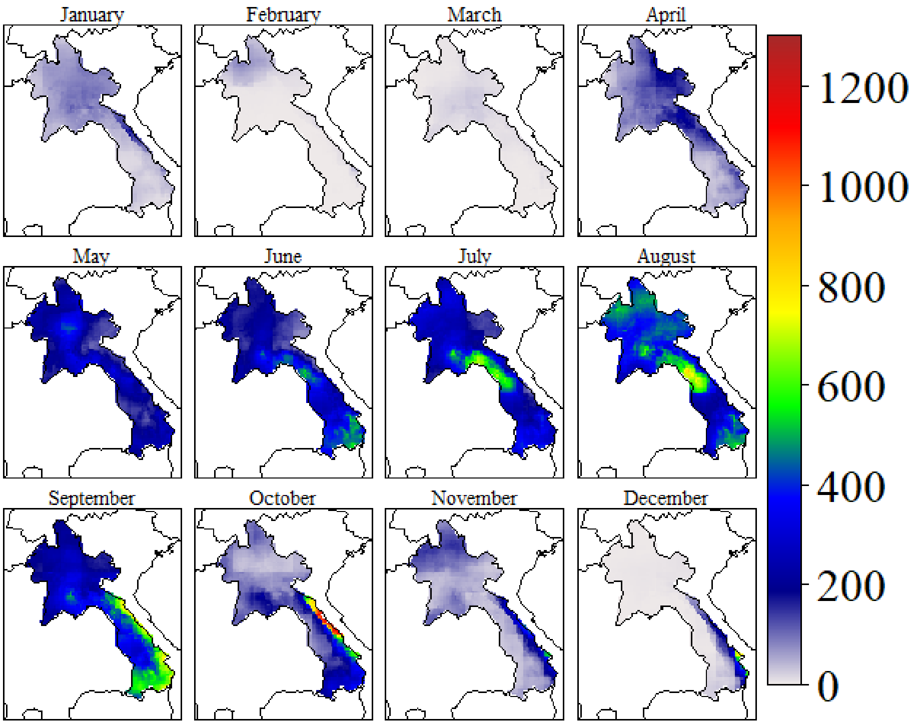

Figure S1: Spatial distribution of the cumulated rainfall (mm month^{-1}) during 2016 over Lao PDR, obtained from the Multi-Source Weighted-Ensemble Precipitation (MSWEP V2) data. Maps were generated using the "raster" package (https://rspatial.org/raster/) and "maptools" package (http://maptools.r-forge.r-project.org/) implemented in R (version 1.2.1335; http://www.r-project.org).
